# Identification of allelic relationship and translocation region among chromosomal translocation lines that leads to less-seed watermelon

**DOI:** 10.1093/hr/uhae087

**Published:** 2024-04-22

**Authors:** Di Jiao, Hong Zhao, Honghe Sun, Jie Zhang, Haiying Zhang, Guoyi Gong, Muhammad Anees, Hongju Zhu, Wenge Liu, Yong Xu

**Affiliations:** National Key Laboratory for Germplasm Innovation & Utilization of Horticultural Crops, Zhengzhou Fruit Research Institute, Chinese Academy of Agricultural Sciences, Hanghai East Road, Guancheng District, Zhengzhou, Henan 450009, China; State Key Laboratory of Vegetable Biobreeding, Tianjin Academy of Agriculture Sciences, Jinjing Road, Xiqing District, Tianjin 300192, China; State Key Laboratory of Vegetable Biobreeding, National Engineering Research Center for Vegetables, Beijing Key Laboratory of Vegetable Germplasms Improvement, Beijing Vegetable Research Center, Beijing Academy of Agriculture and Forestry Science, Zhanghua Road, Haidian Districk, Beijing 100097, China; Plant Biology Section, School of Integrative Plant Science, Cornell University, 236 Tower Road, Ithaca, New York 14853, USA; Boyce Thompson Institute, 533 Tower Road, Ithaca, New York 14853, USA; State Key Laboratory of Vegetable Biobreeding, National Engineering Research Center for Vegetables, Beijing Key Laboratory of Vegetable Germplasms Improvement, Beijing Vegetable Research Center, Beijing Academy of Agriculture and Forestry Science, Zhanghua Road, Haidian Districk, Beijing 100097, China; State Key Laboratory of Vegetable Biobreeding, National Engineering Research Center for Vegetables, Beijing Key Laboratory of Vegetable Germplasms Improvement, Beijing Vegetable Research Center, Beijing Academy of Agriculture and Forestry Science, Zhanghua Road, Haidian Districk, Beijing 100097, China; State Key Laboratory of Vegetable Biobreeding, National Engineering Research Center for Vegetables, Beijing Key Laboratory of Vegetable Germplasms Improvement, Beijing Vegetable Research Center, Beijing Academy of Agriculture and Forestry Science, Zhanghua Road, Haidian Districk, Beijing 100097, China; National Key Laboratory for Germplasm Innovation & Utilization of Horticultural Crops, Zhengzhou Fruit Research Institute, Chinese Academy of Agricultural Sciences, Hanghai East Road, Guancheng District, Zhengzhou, Henan 450009, China; National Key Laboratory for Germplasm Innovation & Utilization of Horticultural Crops, Zhengzhou Fruit Research Institute, Chinese Academy of Agricultural Sciences, Hanghai East Road, Guancheng District, Zhengzhou, Henan 450009, China; National Key Laboratory for Germplasm Innovation & Utilization of Horticultural Crops, Zhengzhou Fruit Research Institute, Chinese Academy of Agricultural Sciences, Hanghai East Road, Guancheng District, Zhengzhou, Henan 450009, China; State Key Laboratory of Vegetable Biobreeding, National Engineering Research Center for Vegetables, Beijing Key Laboratory of Vegetable Germplasms Improvement, Beijing Vegetable Research Center, Beijing Academy of Agriculture and Forestry Science, Zhanghua Road, Haidian Districk, Beijing 100097, China

## Abstract

Less-seed and seedless traits are desirable characteristics in watermelon (*Citrullus lanatus*). Hybridization between watermelon chromosomal translocated lines and wild lines significantly reduced seed counts in the hybrid fruits, approaching even seedless. However, the allelic relationships and the chromosomal translocation breakpoints from different sources are unclear, which limits their utility in breeding practices. This study focused on three groups of chromosomal translocation materials from different sources and conducted inheritance and allelic relationship analysis of translocation points. The results from third-generation genome sequencing and fluorescence in situ hybridization (FISH) revealed that the specific translocations in the naturally mutated material MT-a involved reciprocal translocations between Chr6 and Chr10. The Co^60^γ radiation-induced mutant material MT-b involved reciprocal translocations between Chr1 and Chr5, Chr4 and Chr8. The Co^60^γ radiation-induced mutant material MT-c involved complex translocations among Chr1, Chr5, and Chr11. Cytological observation showed that heterozygous translocation hybrids showed chromosomal synapsis abnormalities during meiotic diakinesis. Further, dominant and codominant molecular markers were developed on both sides of the translocation breakpoints, which could facilitate rapid and efficient identification of chromosome translocation lines. This study provides technical guidance for utilizing chromosomal translocation materials in the development of less-seed watermelon varieties.

## Introduction

Seedless watermelons have gained immense popularity among consumers due to their exceptional qualities, making them a primary focus in watermelon breeding and production. In the 1950s, the triploid seedless watermelons were rapidly promoted by artificially induced tetraploid watermelon plants using colchicine and crossed with diploid watermelon [1]. Triploid seedless watermelon varieties became widely adopted in the USA, South America, and Europe, dominating watermelon production [2]. However, in recent years, the production of triploid seedless watermelons has declined in East Asia, particularly in China. Despite significant improvements in breeding and seed propagation techniques for triploid seedless watermelons, several production challenges persist in seedless watermelon production [3]. These challenges include low seed production, low germination rates, poor seedling establishment, and suboptimal fruit sets.

Additionally, in the 1980s, the introduction of the plant growth regulator CPPU facilitated the induction of parthenocarpy in seedless or less-seed watermelons, while posing potential risks in green watermelon production [4]. Recently, the creation of less-seed watermelon traits through the use of chromosomal translocation materials has introduced a new direction in breeding seedless or less-seed watermelons. This technique does not rely on exogenous growth regulators and overcomes the challenges faced by triploid seedless watermelon production. In China, the selection and application of translocation less-seed watermelon began in the early 1970. A series of diploid heterozygous translocation less-seed watermelon varieties, such as “less-seed Xiaofeng”, “Yihong No. 1” and “Jinhua No. 4” were cultivated successively. The popularization and development of translocation less-seed watermelons enriched the watermelon market.

The less-seed trait is produced by crossing a diploid translocation line with a diploid wild type. During the first meiosis, chromosomal pairing occurs between translocated and normal chromosomes. Subsequent unequal chromosome segregation leads to the loss of genetic material in some gametes, causing the partial abortion of ovules and pollen and resulting in the less-seed phenotype [5]. Previous studies on the creation of translocation materials have utilized X-ray radiation to induce mutagenesis to create unsuitable translocation materials. F_1_ hybrids of translocation lines and normal wild types lead to the formation of “ring” or “chain” of different chromosomes [6, 7]. Some other researchers have used different doses of Co^60^γ radiations on diploid watermelon seeds and demonstrated the induction of chromosomal translocations [8]. There are also naturally occurring translocation materials. For example, a study in 2021 identified a chromosomal breakpoint within a 2.09-Mb region on Chr6 in a natural, spontaneous watermelon line showing the less-seed trait. The hybrid translocation individuals showed abnormal chromosomal segregation during meiosis, resulting in the less-seed phenotype [9]. However, in the artificially induced translocation lines and the naturally existing mutations, the specific recombination band type and the exact translocation relationship remain unclear.

Previous studies on translocation materials have not explained the underlying mechanism of translocation that induces less-seed traits. Researchers have described the less-seed traits resulting from chromosomal translocations, but the exact mechanisms behind these traits remain unknown. Initially, G-band karyotyping was used [10], followed by the widespread adoption of fluorescence in situ hybridization (FISH) [11] which allows the cytological validation and chromosome-level localization of translocation points. Advancements in third-generation sequencing technologies, especially long-read sequencing (LRS) methods, have significantly enhanced the detection of genomic structural variations. LRS, particularly after the advent of HiFi reads, has greatly improved accuracy, enabling studies in pan-genomics [12], complex genome structural variations [13], and chromosome rearrangement detections [14].

This study aimed to elucidate the genetic relationships of allelic variations by crossing three different translocation source materials with normal materials and observing the pollen abortion, ovule development, seed counts in fruits, and progeny segregation in the F_1_ generation. Furthermore, third-generation genome sequencing was used to sequence and assemble the genomes of the three translocation materials and related wild types. PCR sequencing and FISH were used to identify the translocation regions. Cytological observation was used to confirm abnormal chromosomal synapsis during diakinesis in heterozygous translocation materials. Molecular markers developed on either side of the translocation breakpoints enhanced the identification and breeding efficiency of chromosomal translocation lines, laying the theoretical groundwork for future artificial chromosome engineering in watermelons.

## Results

### The fruits of heterozygous F1 hybrids displayed less-seed phenotype

**Figure 1:**
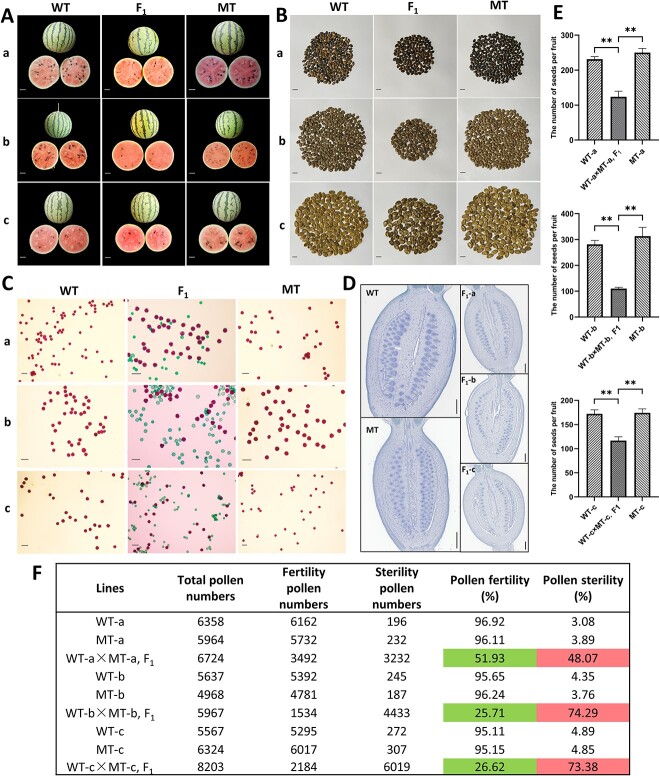
Seed fertility phenotypic analysis of WT (a, b, c), MT (a, b, c), and F_1_ (a, b, c). (A) Fruits and cross sections of WT (a, b, c), MT (a, b, c), and F_1_ (a, b, c). Scale bars = 3 cm. (B) Mature seeds in single fruit of WT (a, b, c), MT (a, b, c), and F_1_ (a, b, c). Scale bars = 1 cm. (C) Alexander’s staining of pollen grains in WT (a, b, c), MT (a, b, c), and F_1_ (a, b, c). Scale bars = 100 μm. (D) Ovary longitudinal sections in WT (a, b, c), MT (a, b, c), and F_1_ (a, b, c). Scale bars = 0.5 mm. (E) The seed numbers of single fruit in WT (a, b, c), MT (a, b, c), and F_1_ (a, b, c). Values represented the mean of three replicates ± SE. The statistical data were analyzed with a one-tailed Student’s *t* test to evaluate significance. ^**^P < 0.01. (F) Statistical analysis of the pollen abortion phenotypes. Green and red marks showed F_1_(a, b, c) pollen have different rates of abortion.

When homozygous translocation lines were hybridized with homozygous normal self-crossed lines, there was a significant reduction in the number of seeds in heterozygous F_1_ hybrids. To further investigate the impact on male and female gametes, we used the homozygous translocation line “Zhongyo-10” as the translocation chromosome donor parent and the homozygous wild-type material WT-a as the recurrent parent to produce the homozygous translocation near-isogenic line MT-a by continuous backcrossing. Moreover, using the homozygous translocation line “Huazhi A” as the translocation donor parent and the homozygous wild-type WT-b,WT-c as the recurrent parents, we developed the homozygous translocation near-isogenic lines MT-b and MT-c. We hybridized homozygous wild types (WT-a, WT-b, WT-c) with homozygous translocation lines (MT-a, MT-b, MT-c) to obtain the heterozygous translocation lines F_1_-a (WT-a × MT-a), F_1_-b (WT-b × MT-b), and F_1_-c (WT-c × MT-c) (Fig. 1A). The phenotype of female gametophytes (unfertilized ovules, seeds after fertilization) and male gametophytes (pollen grains) of the parental materials and their F_1_ generations were observed. The probability of female and male gamete sterility was statistically analyzed.

The average seed numbers per fruit were analyzed statistically. The average seed number of single fruit of WT-a was 231, and MT-a was 250, with no significant difference. In comparison, the average seed number of a single fruit in their F_1_ (F_1_-a) was 123. In F_1_-a, the seed quantity decreased by an average of 48.51% compared to the parental lines (WT-a and MT-a) with a decrease of 46.5% and 50.52%, respectively. The seed number of a single fruit in WT-b was 281, and in MT-b was 313. In contrast, the average seed number of F_1_-b was 110. In F_1_-b, the seed quantity decreased by an average of 62.90% compared to the parental lines WT-b (60.91%) and MT-b (64.88%). Similarly, the average seed number of single fruit of WT-c and MT-c was 172 and 174, respectively, with no significant difference. But the average seed number of their F_1_ (F_1_-c) fruit was 116. In F_1_-c, the seed quantity was decreased by an average of 32.56% compared to the parental lines WT-c (32.17%) and MT-c (32.95%) (Fig. 1B, E).

Alexander staining of mature pollen grains of homozygous wild types (WT-a, WT-b, WT-c), homozygous translocation lines (MT-a, MT-b, MT-c), and their hybrids (F_1_-a, F_1_-b, F_1_-c) revealed that the fertile pollen grains developed red colors, while sterile pollen grains developed green (Fig. 1C). Statistical analysis showed that over 95% of the pollen grains from the three wild types and homozygous translocation line displayed a red color, indicating normal morphology, with less than 5% of sterile pollen grains. In contrast, the three heterozygous translocation lines, F_1_-a, F_1_-b, and F_1_-c exhibited a pollen sterility rate of 48.07%, 74.29%, and 73.38%, respectively (Table 1).

**Table 1:** The pollen abortion isolation in F_2_ population

F_2_ line	Plants number	Normal pollen	Sterility pollen	χ^2^	Mendelian segregation
WT-a × MT-a	237	112 (47.3%)	125 (52.7%)	0.713	1:1
WT-b × MT-b	182	46 (25.3%)	136 (74.7%)	0.007	1:3
WT-c × MT-c	173	65 (37.6%)	108 (62.4%)	14.583	1:3

The χ^2^ test was used to test for quasi-Mendelian segregation of the pollen abortion. χ^2^_0.05，1_ = 3.84.

Histological observations of unfertilized ovules from female flowers opened on the same day for homozygous wild types (WT-a, WT-b, WT-c), homozygous translocation lines (MT-a, MT-b, MT-c), and their hybrids (F_1_-a, F_1_-b, F_1_-c) materials showed a comparison of the number of intact ovules in a single longitudinal section. Both homozygous wild types and homozygous translocated lines showed an average of 70–90 intact ovules. However, in the three heterozygous translocation lines, the average number of intact ovules in a single longitudinal section ranged from 25 to 65 (Fig. 1D). Further, examination of longitudinal sections of ovules revealed a significant reduction in the number of ovules in translocated heterozygous lines compared to the parental lines.

Phenotypic analysis of the number of seeds, pollen grains, and unfertilized ovules indicated a normal development in homozygous wild types and homozygous translocation lines, while the heterozygous translocation lines showed a significant reduction in the average number of seeds, pollen viability, and the number of ovules compared to the parental lines.

### Allelic relationship and inheritance pattern of three translocation materials

**Figure 2:**
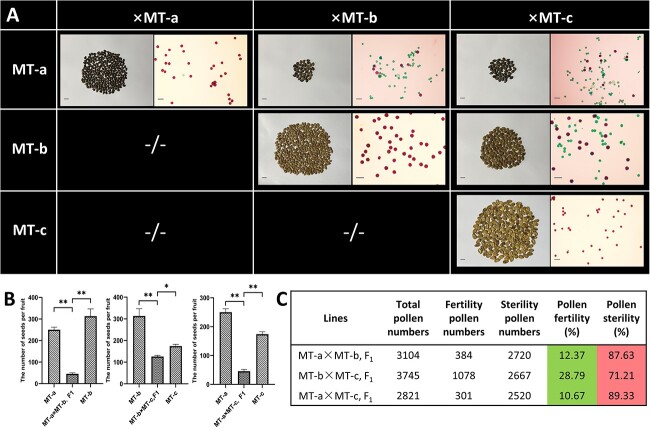
Allelic analysis among MT-a, MT-b, MT-c, and statistical analysis of the pollen abortion phenotype. (A) Mature seeds and pollen fertility of MT (a, b, c) and F_1_(MT-a × MT-b), F_1_(MT-a × MT-c), F_1_(MT-b × MT-c). Scale bars of mature seeds and pollen fertility were 1 cm and 100 μm. (B) The single fruit seed number of MT (a, b, c) and F_1_(MT-a × MT-b), F_1_(MT-a × MT-c), F_1_(MT-b × MT-c). Values represented the mean of three replicates ± SE. The statistical data were analyzed with a one-tailed Student’s *t* test to evaluate significance. ^*^P < 0.05, ^**^P < 0.01. (C) Statistical analysis of the pollen abortion phenotypes. Green and red marks showed F_1_(a, b, c) pollen have different rates of abortion.

To identify the allelic relationship among these three translocated materials (MT-a, MT-b, MT-c), we performed pairwise crossing experiments among the three translocation lines (MT-a, MT-b, MT-c) (Fig. 2A). The results showed that the F_1_ pollen sterility rate of MT-a × MT-b reached 87.63%, with an average seed reduction of 83.61%, MT-b × MT-c resulted in a pollen sterility rate of 71.21% with an average seed reduction of 43.81%, and MT-a × MT-c resulted in a pollen sterility rate of 89.33% with an average seed reduction of 77.80% compared to the parents on average (Fig. 2B, C). These results revealed a less-seed phenotype in all three hybrid combinations of F_1_, indicating no allelic relationship among the three translocated materials belonging to different sources of translocated mutants.

To explore the inheritance patterns of less-seed phenotypes in heterozygous translocated lines from different sources, we statistically analyzed the pollen sterility in F_2_ populations. In WT-a × MT-a, F_2_ populations, the ratio of normal pollen to sterile pollen was 112:125. Upon χ^2^ test, it conformed to a 1:1 Mendelian segregation ratio. In WT-b × MT-b, F_2_ populations, the ratio of normal pollen to sterile pollen was 46:136. Upon χ^2^ test, it conformed to a 1:3 Mendelian segregation ratio. Similarly, for WT-c × MT-c, F_2_ populations, the ratio of normal pollen to sterile pollen was 65:108, but upon χ^2^ test, it was inconsistent with a 1:3 Mendelian segregation ratio. Further, the WT-c × MT-c, F_2_ segregating population showed distorted Mendelian segregation (Table 1).

### Identification of translocated regions based on third-generation genome sequencing

**Figure 3:**
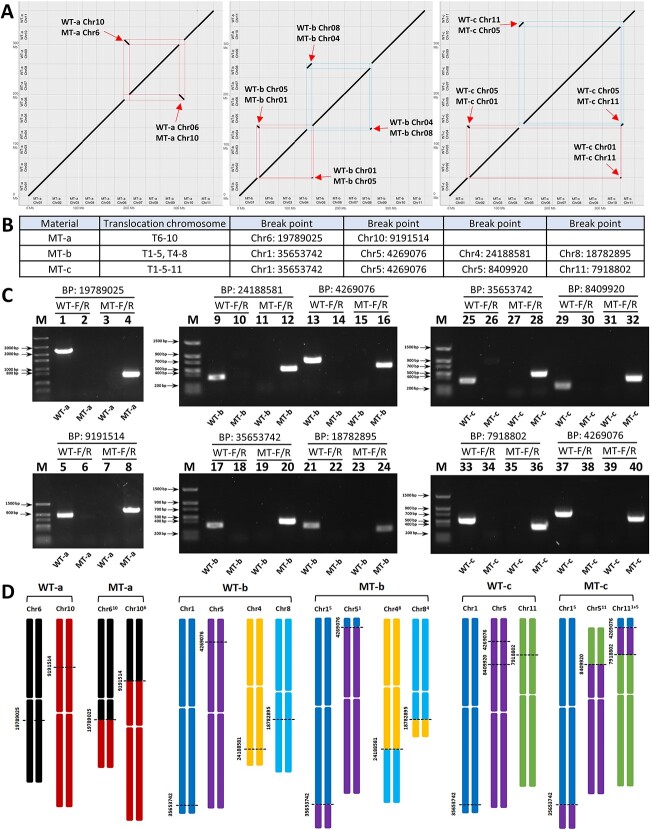
Genome sequencing and translocation region validation. (A) Genome alignment of WT (a, b, c) and MT (a, b, c). Red arrows and red/blue boxes show the same chromosomal translocation region as WT and MT. (B) Chromosomal translocation breakpoint specific location in MT (a, b, c). (C) PCR verification of chromosomal translocation using translocation breakpoints. (1–8) PCR amplification results of WT-a and MT-a in break point: 19789025, 9 191 514 amplification of nonrecombinant primer (WT-F/R) and recombinant primer (MT-F/R). (9–24) PCR amplification results of WT-b and MT-b in break point: 24188581,4 269 076, 35 653 742, 18 782 895 amplification of non-recombinant primer (WT-F/R) and recombinant primer (MT-F/R). (25–40) PCR amplification results of WT-c and MT-c in break point: 35653742, 8 409 920, 7 918 802, 4 269 076 amplification of non-recombinant primer (WT-F/R) and recombinant primer (MT-F/R). (D) Diagram of chromosomal translocation occurrence pattern, with numbers representing the physical location of chromosomal translocation breakpoints.

The genomes of three wild-type materials (WT-a, WT-b, WT-c) and three translocated mutant materials (MT-a, MT-b, MT-c) were sequenced using Pacific Biosciences (PacBio) sequencing, resulting in 9.07 ~ 15.82 G bps HiFi reads, representing 21.1 ~ 36.8× coverage of their genomes. Chromosome-scale assemblies were obtained for each genome, with an assembly size of 370.88 ~ 373.48 Mb, contig N50 length of 27.99 ~ 32.47 Mb, and an average of 99.3% sequences were anchored to the 11 pseudochromosomes, with an average of 1.7 contigs per chromosome. The base quality was ~66 on average, and the genome completeness was estimated as 99.2%–99.4% by Merqury and around 99.1% by BUSCO analysis. Taken together, the high-quality, chromosome-scale genome assemblies assured the accurate identification of the inter-chromosome translocation. (Supplementary Table S1). Chromosomal alignment analysis was conducted between WT-a vs MT-a, WT-b vs MT-b, and WT-c vs MT-c. The results revealed two pairs of chromosomes, Chr6 and Chr10, undergoing translocation in MT-a (which we denoted as Chr6^10^ and Chr10^6^). In MT-b, Chr1 and Chr5 (Chr1^5^, Chr5^1^), Chr4 and Chr8 (Chr4^8^, Chr8^4^), two sets of two chromosomes undergoing translocation were identified. In MT-c, and Chr1 and Chr5 (Chr1^5^, Chr5^1^), Chr5 and Chr11 (Chr11^5’^ and Chr5’^11^) undergoing translocation were identified, suggesting a potential continuous composite translocation involving in Chr1, Chr5, and Chr11 (Fig. 3A). The specific translocation regions are detailed in the figure (Fig. 3B).

Based on the sequencing results, we identified the chromosomal translocation regions and designed specific PCR primers on both sides of the nonrecombinant and translocated recombinant chromosome translocation breakpoints (Supplementary Table S2; Supplementary Fig. S1). Subsequently, the translocation breakpoints were validated using PCR-based amplification. The genome sequencing results revealed two pairs of chromosomes, Chr6 and Chr10, undergoing translocation in MT-a (which we denoted as Chr6^10^ and Chr10^6^). Chr1 and Chr5, Chr4 and Chr8, undergoing translocation in MT-b (which we denoted as Chr1^5^ and Chr5^1^, Chr4^8^ and Chr8^4^). MT-c contained Chr1 and Chr5, Chr5 and Chr11 undergoing translocation (which we denoted as Chr1^5^ and Chr5^1^; Chr11^5^ and Chr5^11^) (Fig. 3C). Further, the sequence amplification of the recombinant chromosomes confirmed the occurrence of chromosomal translocation events. Chromosomal translocations of the three translocation lines are shown in the figure (Fig. 3D).

By comparing the sequence information on both sides of the translocation breakpoints between homozygous wild-type and homozygous translocation mutants, it was found that WT-a had a 5′-Nucleotid (5 nt) micro-homologous sequence (TTTAT) on one side of the Chr6 and Chr10 breakpoints. Post-translocation, MT-a showed 3 nt deletions in recombinant chromosomes Chr6^10^ and Chr10^6^. MT-b exhibited a 25 nt deletion in recombinant chromosome Chr1^5^, 24 nt deletion in Chr5^1^, 4 nt deletion in Chr4^8^, and a 4 nt deletion in Chr8^4^ after translocation. MT-c displayed a 25 nt deletion in recombinant chromosome Chr1^5^, 24 nt deletion in Chr5^1^, 1 nt deletion in Chr5^11^, and a 4 nt deletion in Chr11^5^ after translocation. Sequence alignment results indicated 1-25 bp (base pair) deletions in the recombinant sequences at the translocation breakpoints of both naturally mutated MT-a and radiation-induced MT-b and MT-c translocations. While MT-a had a micro-homologous sequence on one side of the translocation breakpoint, the sequences at the junctions of MT-b and MT-c did not match in a sticky-end manner after the alignment of homologous sequences. Based on the sequence information, it was inferred that the sequence connections at the breakpoint locations were all formed by flat-end joining (Supplementary Fig. S2).

### Cytological verification of chromosomal translocations

**Figure 4:**
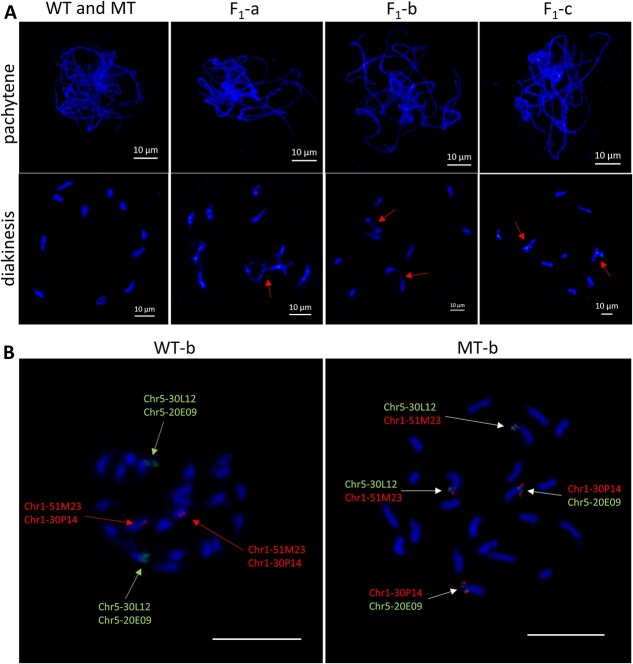
Cytological observation and FISH verification of chromosomal translocation. (A) Cytological behavior of chromosomes during meiosis in WT (a, b, c), MT (a, b, c), and F_1_(a, b, c). Abnormal chromosome coupling dissociation occurs during diakinesis. The arrow indicates the conformation of the chromosome where the abnormal behavior occurs. Scale bars = 10 μm. (B) FISH analysis of WT-b and MT-b. The BAC 51 M23 and 30P14 stained with red fluorescence were located at Chr 1. The BAC 30 L12 and 20E09 stained with green fluorescence were located at Chr 5. In MT-b, BAC 30 L12 and 51 M23 were located at Chr 5^1^, and BAC 30P14 and 20E09 were located at Chr 1^5^. Scale bars = 10 μm.

To further validate the translocation pattern in three different sources of less-seed mutant translocations. The chromosomal morphology during pollen mother cell meiosis of homozygous wild-type (WT- a, b, c), homozygous translocation mutants (MT-a, b, c), and heterozygous translocation mutants (F_1_-a, F_1_-b, F_1_-c) was evaluated. The result showed that the chromosomal morphology and behavior were normal at every stage of the first meiosis in three wild-type materials and three translocation mutant materials. All chromosomal displayed 11 bivalents at diakinesis (Fig. 4A). The heterozygous translocation mutants F_1_-a displayed 9 bivalents and 1 quadrivalents at diakinesis. Two pairs of chromosomes showed intertwined configurations, which formed a “ring 4”. (Fig. 4A). Heterozygous translocation mutants F_1_-b displayed seven bivalents and two quadrivalents at diakinesis, with two pairs of two chromosomes connected in a chain-like pattren (Fig. 4A). Similarly, heterozygous translocation mutants F_1_-c displayed seven bivalents and two quadrivalents at diakinesis, with two pairs of two chromosomes connected and forming two “ring 4” (Fig. 4A). The results of cytological observations indicated abnormal chromosomal synapsis during diakinesis in the three heterozygous translocation materials.

**Figure 5:**
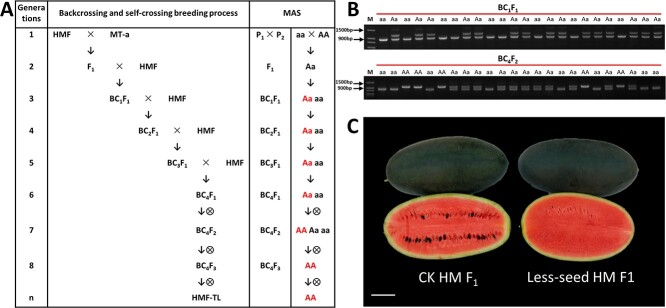
The breeding process of MAS for backcrossing to introduce translocation chromosome. (A) HMF was the backcross parent variety. The red text presented the heterozygous translocation line (Aa) and homozygous translocation line (AA) by MAS in each generation. (B) Heterozygous translocation lines (Aa) were screened on the BC_1_ population using the translocation recombination marker Chr10 + Chr10^6^; Homozygous translocation lines (AA) were screened on the BC_4_F_2_ population. (C) Wild-type HMM and homozygous translocation line HMF-TL were used to prepare the hybrid combination “Less-seed HM F_1_”. Compared with “CK HM F_1_”, which used wild-type “HMM” and wild-type “HMF” as the paternal parent to prepare the hybrid group, the fruit transection section seed number ratio of “Less-seed HM F_1_” was significantly reduced.

Using a BAC library constructed from the watermelon genome, FISH was employed for cytological validation of chromosomal translocations. Specific probes were selected (Supplementary Table S3) based on the genomic sequence inside and outside the translocation breakpoints [26]. As shown in Fig. 5, in the wild-type WT-b, the signals of BAC 51 M23 and BAC 30P14 were co-located on Chr 1, while BAC 30 L12 and BAC 20E09 signals were co-located on Chr 5. However, in the translocation mutant MT-b, BAC 30 L12 and BAC 51 M23 were positioned on the same chromosome, whereas BAC 30P14 and BAC 20E09 were on another chromosomes. Therefore, FISH analysis indicated a mutual translocation between Chr 1 and Chr 5 in MT-b (Fig. 4B).

### Verification and application of chromosomal translocation recombinant markers in the F_2_ population

Chromosomal translocated heterozygous plants exhibit a less-seed phenotype. Thus, the less-seed phenotype caused by chromosomal translocation can be considered a dominant gene in genetics. Based on the third-generation genome sequencing results, we obtained the translocation breakpoints. Through multiplex PCR amplification of nonrecombinant and translocated recombinant chromosome breakpoints, we were able to differentiate between chromosomal recombination/nonrecombination types. Therefore, these differentiations can be used as dominant/codominant markers to detect whether translocation has occurred and is linked to the heterozygous dominant less-seed trait in breeding practices.

To validate the marker accuracy, we used the F_2_ population for genotype and phenotype verification. We conducted marker detection analysis on 237 individual plants of WT-a × MT-a in the F_2_ population. The ratio of homozygous wild-type to heterozygous translocation and homozygous translocation was 1:2:1 upon χ^2^ test, which was consistent with Mendelian segregation ratio. Further, the genotype and phenotype were completely linked and co-segregated (Table 2). In WT-b × MT-b with 182 individual plants in the F_2_ population, the ratio of homozygous wild-type: heterozygous translocation: homozygous translocation upon χ^2^ test confirmed to 1:2:1 Mendelian segregation ratio, a 99.45% genotype–phenotype fit in individuals (Table 2). Similarly, the WT-c × MT-c 173 individual plants in the F_2_ population showed a 1:2:1 segregation ratio upon χ^2^ test analysis, a 99.42% genotype–phenotype fit in individuals (Table 2). These results collectively confirmed that nearly all heterozygous recombinant types detected by the markers underwent pollen sterility, which evidenced a close linkage between chromosomal translocation recombinant markers and the less-seed phenotype in watermelon.

**Table 2:** Translocation recombination markers were verified in F_2_ generation population

F_2_ line	Translocation chromosome	Codominant molecular marker	Plants number	Homozygous WT	Heterozygous TL	Homozygous TL	χ^2^	Mendelian segregation
WT-a × MT-a	T6–10	Chr6 + Chr6^10^	237	51 (21.5%)	125 (52.7%)	61 (25.7%)	1.557	1:2:1
WT-a × MT-a	T6–10	Chr10 + Chr10^6^	237	51 (21.5%)	125 (52.7%)	61 (25.7%)	1.557	1:2:1
WT-b × MT-b	T1–5 + T4–8	Chr1 + Chr1^5^	182	52 (28.5%)	89 (48.9%)	41 (22.5%)	1.417	1:2:1
WT-b × MT-b	T1–5 + T4–8	Chr5 + Chr5^1^	182	49 (26.9%)	91 (50.0%)	42 (23.0%)	0.538	1:2:1
WT-b × MT-b	T1–5 + T4–8	Chr4 + Chr4^8^	182	54 (29.6%)	88 (48.3%)	40 (21.9%)	2.352	1:2:1
WT-b × MT-b	T1–5 + T4–8	Chr8 + Chr8^4^	182	49 (26.9%)	89 (48.9%)	44 (24.1%)	0.363	1:2:1
WT-c × MT-c	T1–5-11	Chr1 + Chr1^5^	173	44 (25.4%)	82 (47.3%)	47 (27.1%)	0.572	1:2:1
WT-c × MT-c	T1–5-11	Chr5 + Chr5^1^	173	44 (25.4%)	82 (47.3%)	47 (27.1%)	0.572	1:2:1
WT-c × MT-c	T1–5-11	Chr5 + Chr5^11^	173	37 (21.3%)	93 (53.7%)	43 (24.8%)	1.393	1:2:1
WT-c × MT-c	T1–5-11	Chr11 + Chr11^5^	173	37 (21.3%)	93 (53.7%)	43 (24.8%)	1.393	1:2:1

The *χ*^2^ test was used to test for quasi-Mendelian segregation of the translocation recombination. χ^2^_0.05，2_ = 5.991

In breeding practices, in order to introduce the translocated chromosome into normal wild-type watermelon varieties, the translocated line MT-a was crossed as the parent with the wild-type material “HMF” which is a East Asian cultivated type materials. The resulting F_1_ hybrids were backcrossed with “HMF” as recurrent parents (RPs) for four generations and then self-crossed (Fig. 5A). Throughout the backcrossing, marker-assisted selection (MAS) was performed using markers for Chr10 non-recombinant type (Chr10-F/Chr10-R) and Chr10 + Chr6 recombinant type (Chr6’-F/Chr10-R) (Fig. 5B). We selected heterozygous translocated recombinant types in the BC_1_–BC_4_ backcrossed generations. Genetically suitable homozygous translocation lines were selected from self-pollinated BC_4_F_2_ progeny base on the molecular markers (Fig. 5B). Eventually, we obtained the homozygous translocation line “HMF-TL”. Utilizing the wild-type “HMM” and homozygous translocation line “HMF-TL” as parental lines, hybrid combinations of “Less-seed HM F_1_” were created. In comparison to hybrid combinations of “CK HM F_1_’created using the wild-type ‘HMM’ and wild-type ‘HMF” as parental lines, the number of seeds in “Less-seed HM-F_1_” was significantly decreased (Fig. 5C).

## Discussion

Utilizing chromosomal translocation lines for the study of less-seed traits in watermelon achieved a significant reduction in seed at the diploid genetic level. Heterozygosis translocation lines could overcome the challenges faced in triploid seedless watermelon production and could hold considerable industrial application value. In traditional translocation line breeding applications, the less-seed trait is heterozygously dominant. Adequate selection of heterozygous translocation materials can be achieved in backcrossed generations through field phenotype observations of pollen sterility and reduced seed in the F_1_ Populations. However, during the selection process of homozygous translocation lines in self-crossed generations, the phenotype of homozygous nontranslocated individuals and homozygous translocated individuals were consistent. Therefore, phenotype selection cannot be performed in the current generation, and it can only be tested through test crossings with normal wild-type hybrids. Thus, the identification of translocation materials to determine specific translocation regions could facilitate the accurate and efficient application of markers in breeding for translocated less-seed traits. Previously, the chromosomal translocations leading to variable traits have been reported in watermelon, including gynoecy [28] and less-seed traits [29]. The identification of the aforementioned translocation lines was conducted through map-based cloning for genetic linkage. In this study, based on three groups of nearly isogenic lines, third-generation LRS was performed. Our results identified naturally occurring and radiation-induced translocation lines and precisely detected the translocated chromosomes and translocation breakpoints. Moreover, designing markers for translocation recombinant types across the translocation breakpoints provided a molecular foundation for MAS-assisted selection of heterozygous translocated less-seed traits. The application of LRS technology could serve as a reference for identifying chromosomal translocations and inversions in other crops [30].

Natural mutation-induced translocation variations are widespread and are possibly influenced by external adverse environmental factors, or the accumulation of reactive oxygen species, which leads to double-strand breaks (DSB) production [31]. Translocations induced by radiation occur due to a series of complex DNA damages caused by ionizing radiation (IR) X-rays or gamma rays (γ-ray) [32], including DSBs, and DSBs on different chromosomes can lead to translocations. However, the translocation occurrence site is generally uncertain. In this study, one type of translocation line was naturally mutated, while the other was radiation-induced, with two completely different induced methods leading to chromosome translocations. Due to varying sources of mutations, it is speculated that natural translocation lines in watermelon from ancestors to artificial domestication may have undergone undetected recombination. The artificially induced heterozygous translocated line “Hua Zhi A” produced offspring MT-b and MT-c with non-allelic relationships. “Hua Zhi A” was initially described as a male sterile line displayed a highly reduced male fertility, as a result of multiple pairs of chromosomal translocations, which led to highly sterile male and female gametes. It can be speculated that the original “Hua Zhi A” heterozygous translocation line may contain all the mutually translocated chromosomes involved in MT-b and MT-c T1–5, T4–8, T5–11. MT-b inherited the independent reciprocal translocation of two pairs of chromosomes in T1–5 and T4–8. According to classical cytology theory, the F1 generation self-cross led to homologous chromosome synapsis formed by two characteristic cross-shaped involving T1–5 and T4–8 at pachytene. Each cross-shaped only produced four fertile gametes by chromosome alternating separation and four abortive gametes by chromosome adjacent separation. The four fertile gamete types combine freely to form 16 zygote types. While the two cross-shaped theoretically produce 1/4 fertile zygote types (homozygous chromosomes) and 3/4 abortive zygote types (heterozygous translocation chromosomes). Therefore, the normal seed ratio in the less-seed phenotype of F_2_ generation is in line with the Mendelian inheritance 1:3. However, a pair of homologous chromosomes in the two reciprocal translocations of MT-c are the same, which may cause the complex translocation of T1–5-11. Since they are not independent reciprocal translocations, in the F_1_ self-crossed generation, the homologous chromosome synapsis formed a complex ring or chain of multiple pairs of chromosomes. Therefore, the F_2_ population did not conform to Mendelian inheritance 1:3.

Endogenous and exogenous factors can cause DSBs, and unrepaired breaks may result in chromosomal translocations. Existing research has proven that many DSB repair mechanisms require the end processing of two DSBs for their connection, leading to modifications or loss of genomic sequences around the breakpoint [33]. In this study, through the analysis of sequences near the breakpoint of the normal wild type and translocation lines. It was found that the natural mutation translocation line MT-a, the radiation mutagenesis translocation line MT-b and MT-c, translocation breakpoint sequences were modified to form different length of base deletion (1-25 bp). The sequence connection form was a flat terminal junction. Therefore, it can be inferred that both natural mutation-induced translocations and radiation-induced chromosomal translocations are forms of direct and rapid non-homologous end repair (NHEJ) [34]. These results were consistent with other reported results of NHEJ as the dominant DSB repair in plants [35].

Radiation mutagenesis plays an important role in the occurrence of free multi-pair recombination of chromosomes and changes in chromosome structure [36]. However, the resulting genomic instability and other undesirable mutations were uncontrollable [37, 38]. Natural mutational translocation has produced stable genomic features through natural and artificial domestication, but nondirected translocation recombination has limited its application in breeding [39]. Human-directed translocations in plants using the CRISPR system have been demonstrated in *Arabidopsis Thaliana* [40], and the extremely low frequency of translocations is still a major problem limiting artificially induced chromosomal translocations. Genetic engineering of Cas9 nucleases [41] or repeated cutting of targeted sites [42] are currently the main research directions for inducing higher frequency translocations. The application of CRISPR technology could provide the possibility for us in the future to induce chromosome breakage and repair, creating artificial translocations, eliminating harmful genes and assembling target genes in a specific way.

## Materials and methods

### Plant materials

Three sets of near-isogenic lines were selected for the current study. The normal-seed wild type (WT-a) was an East Asian cultivated material called “JF”. The translocation mutant type (MT-a) resulted from several backcrosses between WT-a and “Zhongyu 10” (originally from “Jubilee”, a locally bred variety collected by the US National Resources Repository which was a natural mutation translocation line). WT-a and MT-a were near-isogenic lines, which were crossed (WT-a × MT-a) to generate the F_1_ (F_1_-a) and the F_2_ segregation populations (F_2_-a).

Similarly, WT-b, a normal-seed wild type, was an East Asian cultivated variety “JM”. The translocation mutant type MT-b, resulted from several backcrosses between WT-b and “Huazhi A” (an artificially radiation-induced translocation heterozygous mutant obtained by Jinyi Wu of the Guangdong Academy of Agricultural Sciences through Co^60^γ radiation). WT-b and MT-b were near-isogenic lines, which were crossed (WT-b × MT-b) to produce the F_1_ (F_1_-b) and the F_2_ segregation population (F_2_-b).

Finally, WT-c, a normal-seed wild type, was an American cultivated variety “Sugarlee”. The translocation mutant type MT-c resulted from several backcrosses between WT-c and “Huazhi A” (an artificially radiation-induced translocation heterozygous mutant obtained by Jinyi Wu of the Guangdong Academy of Agricultural Sciences through Co^60^γ radiation). Likewise, WT-c and MT-c near-isogenic lines hybrid combination (WT-c × MT-c) produced F_1_ (F_1_-c) and the F_2_ segregation population WT-c × MT-c, F_2_ (F_2_-c).

The watermelon plants were cultivated in the experimental field of the Beijing Academy of Agriculture and Forestry Sciences. In the field, the row-to-row distance of plants was 2 m, and the plant-to-plant distance was 0.3 m. After cutting the side branches, only two main vines were maintained for healthy fruits. Pollination was adopted with the second female flower, using the hand pollination method. The experiment was performed with three biological replicates for each material. The plants were planted continuously for 2 years under natural summer growth conditions.

### Statistics of watermelon fruit seed numbers

The plants were strictly self-pollinated simultaneously under the same growth conditions. The second female flower was selected for each plant to ensure plant growth and fruiting node uniformity. Eight single melons were randomly selected for each material, and the average seed number of single melons was calculated. Data were analyzed in Microsoft Excel 2019 and GraphPad Prism 9.0 with default parameters. Significant differences between groups were determined with one-way analysis of variance and post hoc Tukey’s multiple comparison test.

### Pollen grains Alexander staining

The staining of pollen grains was conducted according to the method as previously reported [15]. Fresh male flowers on the main vines (eight flowers per sample) were carefully selected, and the pollens were collected on slides. Used Alexander staining solution covered for staining for 5–10 minutes, and then observations were made under a microscope. Each male flower was individually prepared on slides, and 5–8 fields were randomly observed. Pollen grain abortion in watermelons was observed and recorded. Viable pollen grains stained purple-red and aborted ones stained green.

### Microscopic examination of ovules

Following the previously reported method [16], ovaries from female flowers on the main vines that bloomed on the same day were fixed in a 50% Formalin-Aceto-Alcohol (FAA) solution for 24 hours. Post-fixation, the samples underwent dehydration in a graded alcohol series, followed by embedding in paraffin. Longitudinal sections of the ovary were prepared, and the paraffin sections were dewaxed for water treatment. Samples were sequentially treated with xylene twice, then socked in anhydrous ethanol, 75% ethanol, and finally rinsed in distilled water. The sections were stained using methylbenzene dye solution and examined under a microscope to differentiate tissue staining levels. Subsequently, the slides were air-dried in an oven, and a transparent coverslip was used for mounting. Microscopic observation and image acquisition were performed for analysis.

### Long read genome sequencing assembly

Plants from each inbred line were grown in a greenhouse until the four-leaf stage and 30 g of fresh leaves were collected for DNA extraction following the PacBio standard protocol. The high-molecular-weight DNA was used to construct SMRTbell libraries for sequencing on the PacBio Sequel II platform using the circular consensus sequencing (CCS) mode, resulting in ~10 Gbp (20X) reads per accession.

The CCS reads were initially processed using the “HiFiAdapter Filt” software to remove adapter sequences [17]. Subsequently, “hifiasm” was used for contig assembly with parameters “-l 2-f 0-u” [18], followed by the removal of redundant sequences using “purge_dups” [19] software and the elimination of contaminant sequences derived from bacteria, viruses, or organelle genomes based on Blast N alignment against the NCBI Nt/Nr database [20]. Finally, the clean genome assembly sequences were anchored on pseudochromosomes using “RagTag” [21] with the “97 103” genome (version 2) as the reference [22]. The assembled chromosome sequences were aligned with the “97 103” genome using minimap2 to infer chromosomal translocations [23].

### DNA extraction and PCR

For each plant in the P_1_, P_2_, F_1_, and F_2_ populations, 1 g leaves were collected and stored at −80°C before use. Genomic DNA was extracted using the cetyltrimethylammonium bromide (CTAB) method. PCR primers were designed using the Premier 5.0 software. The PCR reaction mixture consisted of 20 μl total volume, including 10 μl of PCR Mix buffer (2x), 2 μl DNA (200 ng/μl), 2 μl primer mix (10 μM), and 6 μl ddH2O. PCR amplification was performed using an Applied Biosystems Veriti™ Dx thermal cycler, with an initial denaturation at 95°C for 5 minutes, followed by 35 cycles of denaturation at 95°C for 30 seconds, annealing at 52–55°C for 30 seconds (depending on the primer), extension at 72°C for 1 minute, and a final extension at 72°C for 7 minutes. PCR products were analyzed using 1% agarose gel electrophoresis.

### Chromosome DAPI staining and observation

Following the methods described by Preeda [24], chromosomes were identified by 4′,6-diamidino-2-phenylindole (DAPI) staining. Flower buds from the main shoot of the plant, ranging in diameter from 1.6 to 2.4 mm [25], were collected. The flower buds were fixed in Carnoy fixative (3:1 ethanol: acetic acid) for about 24 hours. After fixation, the flower buds were washed with distilled water several times. Anther were fixed using an enzyme solution consisting of 0.8% pectolyase and 0.8% cellulase at 37°C for 3 h. After enzymatic hydrolysis, the anther was washed with distilled water three times, added 60% glacial acetic acid, and covered with a coverslip to press it down. The sample was put into the −80°C refrigerator for 10 min, the coverslip was lifted immediately and then dried at room temperature. Subsequently, the cells were stained with DAPI, covered with a slide, and observed under a microscope (ZEISS Imager Z2).

### BAC-FISH for the identification of chromosomal translation

Bacterial Artificial Chromosome (BAC) DNA libraries were constructed using the genomic DNA of the watermelon variety 97 103. Four BAC clones located in the regions associated with chromosomal translocations were selected based on the previous report by Guo et al [26]. BAC DNA was extracted and purified using the Phase Prep BAC DNA Kit. The qualified BAC DNA was labeled with digoxigenin or Biotin Nick Translation Mix according to the instructions. Chromosome slides were prepared from flower buds, and subsequent hybridization was conducted following the method reported by Ren *et al*. [27] with slight modifications. At least 20 somatic metaphase samples were observed under a ZEISS Imager Z2 microscope equipped with fluorescent illumination and appropriate filters for DAPI, fluorescein, and Texas-Red fluorescence. Images were captured using a cooled black and white Charge-Coupled Device (CCD) camera (Axiocam 503 color, ZEISS) and processed with ZEN 2 pro software (ZEISS). Final image adjustments were made using Adobe Photoshop 6.0.

## Acknowledgements

This work was financially supported by the National Natural Science Foundation of China (Grant No. 32330093), the Ministry of Agriculture and Rural Affairs of China (CARS-25), the Scientist Training Program of BAAFS (JKZX202401), the Collaborative Innovation Center of BAAFS (KJCX201907-2), Innovation and Development Program of BVRC (KYCX202401), the National Key Research and Development Program of China (2023YFF1000100), agricultural Science, and Technology Innovation Program (CAAS-ASTIP-2021-ZFRI).

## Author contributions

D.J., J.Z., and Y.X. conceived the project. D.J., H.Z., and H.S. performed the experiments. D.J., H.S., and M.A. analyzed the data and wrote the manuscript. W.L., H.Z., and G.G. guided the experiments. J.Z., H.Z., and Y.X. revised the manuscript.

## Data availability

Raw sequencing data have been deposited in the NCBI BioProject database under the accession number PRJNA1071126.

## Conflict of interest statement

The authors declare no conflicts of interest.

## Supplementary Data

Supplementary data is available at *Horticulture Research* online.

## Supplementary Material

Web_Material_uhae087

## References

[1] Kihara H . Triploid watermelons. Proc Am Soc Hort Sci. 1951;58:217–30

[2] Wijesinghe S , Evans LJ, Kirkland L. et al. A global review of watermelon pollination biology and ecology: the increasing importance of seedless cultivars. Sci Hortic. 2020;271:109493

[3] Jaskani MJ , Kwon SW, Kim DH. Comparative study on vegetative, reproductive and qualitative traits of seven diploid and tetraploid watermelon lines. Euphytica. 2005;145:259–68

[4] Hayata Y , Niimi Y, Iwasaki N. Synthetic cytokinin—1-(2-chloro-4-pyridyl)-3-phenylurea (CPPU)—promotes fruit-set and induces parthenocarpy in watermelon. J Am Soc Hortic Sci. 1995;120:997–1000

[5] Nishimura Y , Sakaguchi S. Studies on reciprocal translocations of chromosomes in watermelon. Bull Fac Agric Niigata Univ. 1960;12:22–9

[6] Oka H , Watanabe T, Nishiyama I. Reciprocal translocation as a new approach to breeding seedless watermelon. Can J Genetics Cytol. 1967;9:482–9

[7] Sakaguchi S , Nishimura Y. Breeding seedless watermelon by using induced chromosome translocation. Jpn Agric Res Q. 1969;4:18–21

[8] Wu J , Wu Y, Xie X. Breeding of chromosome translocation line and F_1_ hybrid with reduced seed in the fruit of watermelon. Chin Cucurbits Veg. 2013;26:1–6

[9] Tian S , Ge J, Ai G. et al. A 2.09 Mb fragment translocation on chromosome 6 causes abnormalities during meiosis and leads to less seed watermelon. Hort Res. 2021;8:256 10.1038/s41438-021-00687-9 PMC8633341 34848689

[10] Yunis JJ , Prakash O. The origin of man: a chromosomal pictorial legacy. Science. 1982;215:1525–30 7063861 10.1126/science.7063861

[11] Szinay D , Wijnker E, van den Berg R. et al. Chromosome evolution in Solanum traced by cross-species BAC-FISH. New Phytol. 2012;195:688–98 22686400 10.1111/j.1469-8137.2012.04195.x

[12] Shi J , Tian Z, Lai J. et al. Plant pan-genomics and its applications. Mol Plant. 2023;16:168–86 36523157 10.1016/j.molp.2022.12.009

[13] Lee JH , Venkatesh J, Jo J. et al. High-quality chromosome-scale genomes facilitate effective identification of large structural variations in hot and sweet peppers. Hort Res. 2022;9:uhac210 10.1093/hr/uhac210 PMC9715575 36467270

[14] Kovaka S , Ou S, Jenike KM. et al. Approaching complete genomes, transcriptomes and epi-omes with accurate long-read sequencing. Nat Methods. 2023;20:12–6 36635537 10.1038/s41592-022-01716-8 PMC10068675

[15] Park GT , Frost JM, Park JS. et al. Nucleoporin MOS7/Nup88 is required for mitosis in gametogenesis and seed development in Arabidopsis. Proc Natl Acad Sci. 2014;111:18393–8 25489100 10.1073/pnas.1421911112 PMC4280575

[16] Endress PK . Angiosperm ovules: diversity, development, evolution. Ann Bot. 2011;107:1465–89 21606056 10.1093/aob/mcr120 PMC3108811

[17] Sim SB , Corpuz RL, Simmonds TJ. et al. HiFiAdapterFilt, a memory efficient read processing pipeline, prevents occurrence of adapter sequence in PacBio HiFi reads and their negative impacts on genome assembly. BMC Genomics. 2022;23:1–7 35193521 10.1186/s12864-022-08375-1 PMC8864876

[18] Cheng H , Concepcion GT, Feng X. et al. Haplotype-resolved de novo assembly using phased assembly graphs with hifiasm. Nat Methods. 2021;18:170–5 33526886 10.1038/s41592-020-01056-5 PMC7961889

[19] Guan D , McCarthy SA, Wood J. et al. Identifying and removing haplotypic duplication in primary genome assemblies. Bioinformatics. 2020;36:2896–8 31971576 10.1093/bioinformatics/btaa025 PMC7203741

[20] Camacho C , Coulouris G, Avagyan V. et al. BLAST+: architecture and applications. BMC bioinformatics. 2009;10:1–9 20003500 10.1186/1471-2105-10-421 PMC2803857

[21] Alonge M , Lebeigle L, Kirsche M. et al. Automated assembly scaffolding using RagTag elevates a new tomato system for high-throughput genome editing. Genome Biol. 2022;23:1–19 36522651 10.1186/s13059-022-02823-7 PMC9753292

[22] Guo S , Zhao S, Sun H. et al. Resequencing of 414 cultivated and wild watermelon accessions identifies selection for fruit quality traits. Nat Genet. 2019;51:1616–23 31676863 10.1038/s41588-019-0518-4

[23] Li H . Minimap2: pairwise alignment for nucleotide sequences. Bioinformatics. 2018;34:3094–100 29750242 10.1093/bioinformatics/bty191 PMC6137996

[24] Preeda N , Yanagi T, Sone K. et al. Chromosome observation method at metaphase and pro-metaphase stages in diploid and octoploid strawberries. Sci Hortic. 2007;114:133–7

[25] Ge J , Zhang Y, Tian S. et al. Observation on meiosis process of watermelon pollen mother cell. Chin Cucurbits Veg. 2020;33:16–22

[26] Guo S , Zhang J, Sun H. et al. The draft genome of watermelon (*Citrullus lanatus*) and resequencing of 20 diverse accessions. Nat Genet. 2013;45:51–8 23179023 10.1038/ng.2470

[27] Ren Y , Zhao H, Kou Q. et al. A high resolution genetic map anchoring scaffolds of the sequenced watermelon genome. PLoS One. 2012;7:e29453 22247776 10.1371/journal.pone.0029453 PMC3256148

[28] Zhang J , Guo S, Ji G. et al. A unique chromosome translocation disrupting ClWIP1 leads to gynoecy in watermelon. Plant J. 2020;101:265–77 31529543 10.1111/tpj.14537

[29] Wang M , Zhang X, Zhang X. Breeding few-seed watermelon(*C. lanatus*) via chromosome reciprocal translocation induced by γ-rays. Acta Horticulturae Sinica. 1988;15:125–30

[30] Wang J , Liu W, Zhu D. et al. Chromosome-scale genome assembly of sweet cherry (Prunus avium L.) cv. Tieton obtained using long-read and hi-C sequencing. Hort Res. 2020;7:122 10.1038/s41438-020-00343-8 PMC7395734 32821405

[31] Manova V , Gruszka D. DNA damage and repair in plants–from models to crops. Front Plant Sci. 2015;6:885 26557130 10.3389/fpls.2015.00885 PMC4617055

[32] Shirley BW , Hanley S, Goodman HM. Effects of ionizing radiation on a plant genome: analysis of two Arabidopsis transparent testa mutations. Plant Cell. 1992;4:333–47 1354004 10.1105/tpc.4.3.333 PMC160133

[33] Iyama T , Wilson DM III. DNA repair mechanisms in dividing and non-dividing cells. DNA repair. 2013;12:620–36 23684800 10.1016/j.dnarep.2013.04.015 PMC3720834

[34] McVey M , Lee SE. MMEJ repair of double-strand breaks (director’s cut): deleted sequences and alternative endings. Trends Genet. 2008;24:529–38 18809224 10.1016/j.tig.2008.08.007 PMC5303623

[35] Puchta H . The repair of double-strand breaks in plants: mechanisms and consequences for genome evolution. J Exp Bot. 2005;56:1–14 15557293 10.1093/jxb/eri025

[36] Parry MAJ , Madgwick PJ, Bayon C. et al. Mutation discovery for crop improvement. J Exp Bot. 2009;60:2817–25 19516074 10.1093/jxb/erp189

[37] Jeggo P , Löbrich M. Radiation-induced DNA damage responses. Radiat Prot Dosim. 2006;122:124–7 10.1093/rpd/ncl495 17351270

[38] Van Vu T , Sung YW, Kim J. et al. Challenges and perspectives in homology-directed gene targeting in monocot plants. Rice. 2019;12:1–29 31858277 10.1186/s12284-019-0355-1 PMC6923311

[39] Van Vu T , Thi Hai Doan D, Kim J. et al. CRISPR/Cas-based precision genome editing via microhomology-mediated end joining. Plant Biotechnol J. 2021;19:230–9 33047464 10.1111/pbi.13490 PMC7868975

[40] Beying N , Schmidt C, Pacher M. et al. CRISPR–Cas9-mediated induction of heritable chromosomal translocations in Arabidopsis. Nature plants. 2020;6:638–45 32451449 10.1038/s41477-020-0663-x

[41] Shou J , Li J, Liu Y. et al. Precise and predictable CRISPR chromosomal rearrangements reveal principles of Cas9-mediated nucleotide insertion. Mol Cell. 2018;71:498–509.e4 30033371 10.1016/j.molcel.2018.06.021

[42] Yin J , Lu R, Xin C. et al. Cas9 exo-endonuclease eliminates chromosomal translocations during genome editing. Nat Commun. 2022;13:1204 35260581 10.1038/s41467-022-28900-w PMC8904484

